# Effect of humic acid on anaerobic digestion of cellulose and xylan in completely stirred tank reactors: inhibitory effect, mitigation of the inhibition and the dynamics of the microbial communities.

**DOI:** 10.1007/s00253-016-8010-x

**Published:** 2016-11-29

**Authors:** Samet Azman, Ahmad F. Khadem, Caroline M. Plugge, Alfons J. M. Stams, Sabina Bec, Grietje Zeeman

**Affiliations:** 1 Laboratory of Microbiology, Wageningen University, Stippeneng 4, 6708 WE Wageningen, The Netherlands; 2 Sub-department of Environmental Technology, Wageningen University, Bornse Weilanden 9, 6708 WG Wageningen, The Netherlands; 3 Faculty of Civil Engineering and Geosciences, Department of Water Management, Section Sanitary Engineering, Delft University of Technology, Stevinweg 1, 2628 CN Delft, The Netherlands

**Keywords:** Hydrolysis, Inhibition, Mitigation, Hydrolytic enzymes, Calcium, Microbial community analyses

## Abstract

Inhibition effect of humic acid (HA) on anaerobic digestion of cellulose and xylan and the mitigation potential of the inhibition were evaluated in controlled fed batch reactors at 30 °C and a hydraulic retention time (HRT) of 20 days. Reactor performances were evaluated by biogas production and metabolite measurements for 220 days. Microbial population dynamics of the reactors were monitored with next-generation 16S rRNA gene sequencing at nine different sampling times. Our results showed that increasing levels of HA inhibited the hydrolysis efficiency of the digestion by 40% and concomitantly reduced the methane yield. Addition of hydrolytic enzymes helped to reverse the negative effects of HA, whereas calcium addition did not reverse HA inhibition. Microbiological analyses showed that the relative abundance of hydrolytic/fermentative bacterial groups such as *Clostridiales*, *Bacteroidales* and *Anaerolineales* was significantly lowered by the presence of HA. HA also affected the archaeal populations. Mostly hydrogenotrophic methanogens were negatively affected by HA. The relative abundance of *Methanobacteriaceae*, *Methanomicrobiales-*WCHA208 and Unassigned *Thermoplasmata* WCHA1-57 were negatively affected by the presence of HA, whereas *Methanosaetacea* was not affected.

**Electronic supplementary material:**

The online version of this article (doi:10.1007/s00253-016-8010-x) contains supplementary material, which is available to authorized users.

## Introduction

Recently, sustainable energy production has drawn great interest. Although there are many sources of sustainable energy (e.g. wind, solar, thermal, etc.), specifically biomass is an attractive energy source due to its high energy potential. Traditional biomass processing is the most common way to produce energy (Kopetz 2013; Lauri et al. 2014; Toka et al. 2014). Globally, approximately 47% of the sustainable energy production is derived from biomass (Sawin et al. 2015). Anaerobic digestion is one of the prominent technologies to conserve energy in biomass as biogas (Appels et al. 2011; Tiwary et al. 2015; van Meerbeek et al., 2015). However, available technologies for anaerobic biomass digestion can only recover around 50% of the potential energy (Liu et al. 2015; Raposo et al. 2012). The reason for the lower energy recovery is mainly related to biodegradability of the biomass and the presence of several inhibitors (Azman et al. 2015a; Chen et al. 2008).

Pretreatment technologies have been extensively studied to improve the biodegradability of the biomass and increase the biogas yield during anaerobic biomass digestion (Hendriks and Zeeman 2009; Zheng et al. 2014). Physical, chemical and biological pretreatments and combinations of these pretreatment methods are generally applied. In many cases, pretreatment has a positive effect on biogas yield. However, inhibitory compounds usually remain within bioreactors and even additional recalcitrant molecules can be produced after the pretreatment (Klinke et al. 2004; Negro et al. 2003). Because of that reason, more insights in the effect of inhibitory compounds on anaerobic digestion is required to achieve a more efficient methane production.

Humic acids (HAs) are inhibitors of anaerobic biomass digestion. HA have a very complex chemical structure that their presence can alter the chemistry of the environment (Davies et al. 2001). HA can be found in several environments as they are formed as a result of biological decay. HA are abundant in soil as well as in natural waters, sewage, leaching sites, anaerobic digesters treating manure and agricultural biomass (Fernandes 2010; Kang et al. 2002; Li et al. 2011).

Although the ecological role of the HA in nature is well documented, there are only few papers that describe the (negative) effects of HA on anaerobic cellulosic biomass hydrolysis (Azman et al. 2015b; Brons et al. 1985; Fernandes et al. 2015). The exact mechanism of HA inhibition on hydrolysis is not known, but binding properties of HA to hydrolytic enzymes are proposed to explain the inhibition (Fernandes et al. 2015). HA may play an important role in the low biogas production within biogas plants in which cellulose and xylan are highly abundant (derived from plant residues and manure) (Vassilev et al. 2010). HA content within anaerobic digesters are not well defined, and HA concentrations can reach up to 1.5% *w*/*w* of total solids in the treatment sludge, manure and energy crops that may cause low conversion efficiencies during anaerobic digestion (Fernandes 2010). Thus, negative effects of HA on anaerobic digestion should be taken away to improve biogas production.

Removal of HA can be an option to overcome the negative effects. Indeed, removal of HA from drinking water treatment plants by membrane filtration systems has been successfully achieved (Ren and Graham 2015). On the other hand, extraction, absorption, ion exchange, coagulation and flocculation processes have been proposed to remove HA from several matrices (Li et al. 2014; Song et al. 2013; Tan 2014). Aforementioned methods are not suitable for anaerobic digesters due to the high solid content of the biomass. Thus, different approaches are needed to reverse the inhibitory effects of HA. Two different approaches have been described to overcome HA inhibition in anaerobic digesters. These are hydrolytic enzyme addition and polyvalent cation addition (Azman et al. 2015b; Brons et al. 1985; Fernandes et al. 2015). Addition of hydrolytic enzymes and polyvalent cations can reduce the active binding sites of the HA. In this way, scavenging of the hydrolytic enzymes by HA might be minimised and microbial conversion can proceed. Although these methods reversed the HA inhibition on anaerobic digestion, all the reported experiments were conducted in batch incubations. Therefore, their application possibilities to large-scale digester operations are still unclear.

This study investigates HA inhibition on anaerobic cellulose and xylan digestion and the mitigation of the HA inhibition with three objectives: firstly, to confirm the HA inhibition in fed batch reactors; secondly, to show the feasibility of calcium and hydrolytic enzyme addition to reverse the inhibitory effects of HA; and thirdly, to investigate the effect of HA on the microbial community. In this scope, we operated five fed batch anaerobic reactors in parallel. One reactor was used as a control reactor (R1), whereas the other reactors were used as test reactors (R2–R5). In the test reactors, increasing levels of HA were applied (R2–R5), while additional treatments of calcium addition (R3), hydrolytic enzyme addition (R4) and combination of hydrolytic enzyme and calcium addition (R5) were applied. Reactor performance and microbial community composition were evaluated for 220 days. Correlations between microbial population dynamics and operational parameters were made to couple reactor performances to microbial population dynamics.

## Material and methods

### Operation of fed batch reactors

In total, five lab-scale anaerobic double-wall reactors (total volume 6 L; working volume 5 L) were operated in parallel. All reactors were inoculated with crushed anaerobic granular sludge which was taken from a full-scale up-flow anaerobic sludge bed (UASB) reactor, treating pulp and paper industry effluents (Industriewater Eerbeek, Eerbeek, The Netherlands). Each reactor was equipped with water jackets that were connected to a water bath, circulating water to the water jackets. Constant temperature was assured for each individual reactor at 30 ± 0.5 °C, and operational pH was kept between 6.8 and 7.2 by adding 5 M NaOH when necessary. Continuous stirring of the reactors was obtained by anchor-type propellers at 100 rpm.

Following the inoculation of the reactors, seed sludge was acclimatised to the reactor environment at 30 °C with 1.8 g volatile solids (VS) L^−1^ day^−1^ organic loading rate (OLR) and a hydraulic retention time (HRT) of 20 days for 190 days. A starch, glucose and volatile fatty acids (VFA) mixture (acetate, propionate and butyrate) was fed for 55 days; after that, the feeding continued with a cellulose and xylan mixture till day 190. After the acclimation period, the experiment was initiated. Five reactors were fed every day for another 220 days with synthetic medium, using cellulose (avicel; PH-101, Fluka, Darmstadt) and beech wood xylan (Roth, Karlsruhe, Germany) at a ratio of 75:25 (*w*/*w*). OLR was kept at 1.8 g VS L^−1^ day^−1^. The feed was mixed with mineral based medium which was previously described (Plugge 2005; Stams et al. 1993), with additional 100 mg L^−1^ Fe_2_(SO_4_)_3_ and omitting reducing agents to maintain a HRT of 20 days throughout the experiment. After the first 30 days (P0) of the reactor operation, HA addition and inhibition mitigation experiments were started when the methane production stabilised. Humic acid (Sigma-Aldrich; CAS number 68131-04-4) addition was initiated, starting from day 30 for four reactors (R2, R3, R4 and R5), while R1 was used as a control reactor. HA was added every day in increasing concentrations 2, 20, 40, 100 and 400 mg L^−1^ for different periods (P1 (days 31–50), P2–P3 (days 50–91), P4 (days 92–125), P5 (days 126–146) and P6 (days 147–173), respectively) until reaching inhibition. Starting from P5, a few drops of silicon oil (Sigma-Aldrich, Darmstadt, Germany) were added to the reactors to prevent foaming, when necessary. After an observed inhibition, HA addition was stabilised (P7–P8; days 173–220) to test whether HA inhibition remained stable. Operation conditions are summarised in Table 1.

**Table 1:** Operational conditions of the reactors and the notation of the time periods that were used in the experiments ad.﻿ = addition

Operation days	R1			R2			R3			R4			R5		
	HA ad. (mg (L day)^−1^)	Ca ad.	Enzyme ad.	HA ad. (mg (L day) ^−1^)	Ca ad.	Enzyme ad.	HA ad. (mg (L day) ^−1^)	Ca ad.	Enzyme ad.	HA ad. (mg (L day) ^−1^)	Ca ad.	Enzyme ad.	HA ad. (mg (L day) ^−1^)	Ca ad.	Enzyme ad.
0–30 (P0)	–	–	–	–	–	–	–	–	–	–	–	–	–	–	–
31–50 (P1)	–	–	–	2	–	–	2	+	–	2	–	+	2	+	+
51–70 (P2)	–	–	–	20	–	–	20	+	–	20	–	+	20	+	+
71–91 (P3)	–	–	–	20	–	–	20	+	–	20	–	+	20	+	+
92–125 (P4)	–	–	–	40	–	–	40	+	–	40	–	+	40	+	+
126–146 (P5)	–	–	–	100	–	–	100	+	–	100	–	+	100	+	+
147–173 (P6)	–	–	–	400	–	–	400	–	–	400	–	+	400	–	+
174–194 (P7)	–	–	–	400	–	–	400	–	–	400	–	–	400	–	–
195–220 (P8)	–	–	–	400	–	–	400	–	–	400	–	–	400	–	–

R3, R4 and R5 were used to test three different mitigation strategies for HA inhibition. CaCl_2_ (Sigma-Aldrich) was added to R3 and R5; 0.11 g CaCl_2_ g HA_added_^−1^ was supplied within different periods (P1, P2, P3, P4 and P5). Three enzyme cocktails were obtained from DSM (Delft, The Netherlands) and were used for the enzyme addition experiments. All enzymes were multicomponent mixtures that had side enzyme activities. The first enzyme was a cellulase, which had cellulase, β-glucanase and xylanase activity. Cellulase was produced by a commercial *Trichoderma* strain and contained 100 mg protein mL^−1^. The second enzyme was an endoglucanase, which had β-glucanase, cellulase and xylanase activity. Endoglucanase was produced by a commercial *Talaromyces* strain and contained 90 mg protein mL^−1^ (suspension was prepared with demi-water at 10 mg protein mL^−1^). The third enzyme was a xylanase which had xylanase and β-glucanase activity. Xylanase was produced by a commercial *Aspergillus* strain and contained 150 mg protein g^−1^. Both enzymes were dosed to the reactors based on protein content. The amount of added enzymes was expressed as milligrammes of protein per HA added to the reactors: 0.6 mg cellulase mg HA_added_^−1^, 0.075 mg xylanase mg HA_added_^−1^ and 0.55 mg endoglucanase mg HA_added_^−1^ for P1 to P5, and the enzyme amount was reduced by half for P6. Enzyme addition was stopped in P7 and P8.

Biogas production was monitored by a gas flow measurement device (μflow, Bioprocess Control, Lund, Sweden) and recorded daily. Cumulative biogas production was recorded daily and expressed in millilitres at standard temperature and pressure (STP), 0 °C, 1 atm). Biogas composition was quantified biweekly via gas Intrscience GC 8000 chromatograph (Interscience, Breda, The Netherlands) equipped with a thermal conductivity detector and two columns (Molsieve 5A 50 m × 0.53 mm (Agilent, Santa Clara, MA) for hydrogen, nitrogen and methane and Porabond Q 50 m × 0.53 mm (Agilent, Santa Clara, MA) for CO_2_. Temperatures of injector, detector and oven were 110, 99 and 50 °C, respectively. Organic acids were quantified using a Thermo Scientific Spectrasystem HPLC system (Thermo Scientific, Waltham, MA), equipped with a Varian Metacarb 67H 300 × 6.5 mm column (Agilent, Santa Clara, MA) kept at 45 °C, running with 0.005 M sulphuric acid as eluent. The eluent had a flow rate of 0.8 mL min^−1^. The detector was a refractive index detector. Data analyses were performed using ChromQuest (Thermo Scientific, Waltham, MA). The total organic acid concentrations were expressed as their chemical oxygen demand (COD) equivalents (mg L^−1^ COD) of measured acetate and propionate concentrations. Hydrolysis, acidogenesis and methanogenesis efficiencies were calculated with the formulas that were described previously (Azman et al. 2015b, Formula S1 in the Supplementary Material). The biological methane potential (BMP: mL CH_4_ mL enzyme mixture^−1^) of the enzyme mixture was measured as described previously (Azman et al. 2015b). Since the methane production from the enzyme mixtures contributed to the total methane yields significantly, methane production in R4 and R5 were corrected for methane that was derived from the enzyme mixtures.

### Microbial community monitoring by next-generation 16S rRNA amplicon sequencing

Fifty millilitres of sludge samples were collected in the beginning and at the end of each period. Samples were kept at −20 °C prior to genomic DNA extraction. Genomic DNA extraction from the nine sampling points (P0 to P8) was performed using Fast DNA® SPIN kit for soil (MP Biomedicals, Solon, OH) following the manufacturer’s protocol with additional washing steps before starting to the extraction. 1X PBS solution with 0.5 mM EDTA was used to wash pellets two times to remove the HA from the solids which could be inhibitory for the PCR reactions. The DNA yields were measured with a Nanodrop® (ND-1000) spectrophotometer (Nanodrop Technologies, Wilmington, DE). DNA qualities were checked using the OD 260/280 ratio. Samples that had 1.80 ± 0.15 260/280 values were considered as good-quality DNAs, and amplicon sequencing was performed with those samples.

Extracted DNA from selected samples was used for bacterial and archaeal community analysis. The amplification of bacterial and archaeal gene fragments was done using a two-step PCR. First amplification of bacterial 16S rRNA gene fragments was done using the 27 F-DegS (5′-GTT[TC]GAT[TC][AC]TGGCTCAG-3′) (van den Bogert et al. 2011, 2013) and equimolar mix of two reverse primers (338R-I and 338-R-II (5′-GC[AT]GCC[AT]CCCGTAGG[TA]GT-3′) (Daims et al. 1999)), and the first amplification of archaeal 16S rRNA gene fragments was done using primers 518F (5′-CAGC[AC]GCCGCGGTAA-3′) (Wang and Qian 2009) and 905R (5′-CCCGCCAATTCCTTTAAGTTTC-3′) (Kvist et al. 2007). PCR amplifications were carried out in technical duplicates in a total volume of 50 μl containing 500 nM of each forward and reverse primer (Biolegio BV, Nijmegen, The Netherlands), 1 unit of Phusion DNA polymerase (Thermo Scientific, Waltham, MA), 10 μl of high-fidelity (HF) buffer, 200 μM dNTP mix and 1 μl DNA template, made to a total volume of 50 μl with nuclease-free sterile water. The PCR program was as follows: denaturing at 98 °C for 30 s, followed by 25 cycles of denaturing at 98 °C for 10 s, annealing at 56 °C for bacterial and 60 °C for archaeal for 20 s, extension at 72 °C for 20 s, followed by a final extension step at 72 °C for 10 min. After positive amplifications, technical duplicates were pooled and prepared for the second step PCR amplification. A second amplification was performed to extend 8 nt barcodes to the amplicons, as described previously (Hamady et al. 2008). Barcoded amplification was performed in a total volume of 100 μl containing 5 μl of the first PCR product, 500 nM of each forward and reverse primer (Biolegio BV, Nijmegen, The Netherlands), 2 units of Phusion DNA polymerase (Thermo Scientific, Waltham, MA), 20 μl of high-fidelity (HF) buffer and 200 μM dNTP mix, made to a total volume of 100 μl with nuclease-free water. The PCR program was as follows: denaturing at 98 °C for 30 s, followed by five cycles of denaturing at 98 °C for 10 s, annealing at 52 °C for 20 s, extension at 72 °C for 20 s, followed by a final extension at 72 °C for 10 min. Barcoded PCR products were cleaned using the HighPrep PCR clean-up system (MagBio Genomics Inc., Gaithersburg, MD). DNA was quantified using Qubit (Invitrogen, Bleiswijk, The Netherlands). After the second PCR, barcoded samples were pooled in equimolar quantities to create a library. The libraries were purified again by using the same purification protocol.Prepared libraries were sent to GATC company (Konstanz, Germany) for Hiseq sequencing on the Illumina platform.

### Sequencing data analysis

16S rRNA gene sequencing data was analysed using NG-Tax, an in-house pipeline (Ramiro-Garcia et al. 2016). Paired-end libraries were filtered to contain only read pairs with perfectly matching barcodes, and those barcodes were used to demultiplex reads by sample. Resulting reads were separated by sample using the affiliated barcodes. Taxonomy affiliation was done with the SILVA 16S rRNA reference database by using an open reference approach as described by Quast et al. (2013). Quantitative Insights into Microbial Ecology (QIIME) v1.2 (Caporaso et al. 2010) was used to define microbial compositions based on the described pipeline. The project was deposited to NCBI’s Sequence Read Archive (SRA) under project number PRJNA320994.

### Statistical analyses

Significant differences between reactor operational parameters were checked with one-way ANOVA test. When the ANOVA rules were matched, post hoc tests (Tukey’s honest significant difference test) were applied to further compare the operational data. Differences were considered statistically significant at a *p* value <0.05, or otherwise stated.

The influence of process parameters on the microbial community composition was analysed using redundancy analyses (RDAs) with the CANOCO software (version 5) (Šmilauer and Lepš 2014). The significance test for RDA was carried out by Monte Carlo permutation (499 times), and correlations were considered significant at a *p* value <0.05. Ranked Spearman correlation was also applied to determine the correlation between microbial groups and operational conditions. All statistical and correlation analyses were performed by IBM SPSS Statistics 23 (Armonk, NY).

## Results

### Anaerobic digester performance

“Steady-state” conditions, in which stable methane production and effluent VFA concentrations were reached (El-Mashad et al. 2004), were achieved before initiation of the HA inhibition experiments with an HRT of 20 days. The complete operation time of the reactors was divided into eight different periods as given in Table 1. Different time periods also reflect the sampling points for the microbiological analyses.

**Fig. 1:**
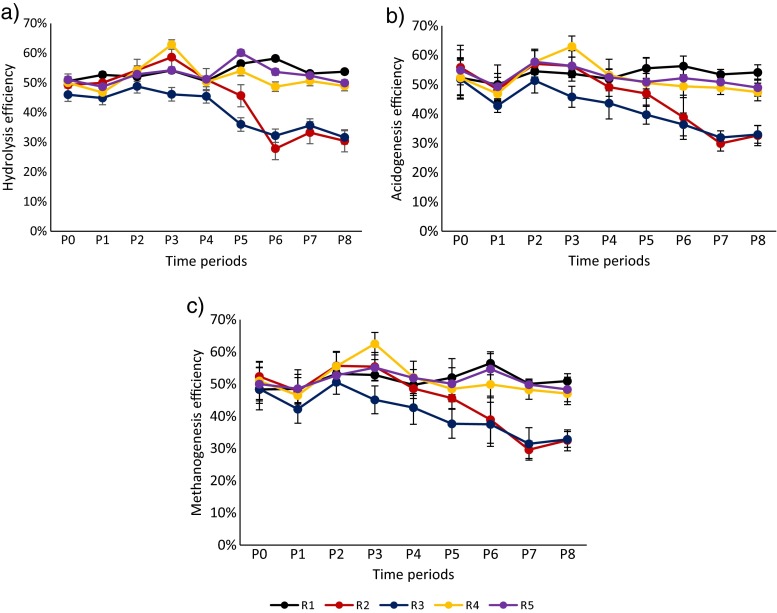
Hydrolysis, acidogenesis and methanogenesis efficiencies (%) of the reactors throughout the experiment. Each data point represents the average efficiencies within the mentioned time period. The reactors were represented with R1 (control), R2 (inhibition), R3 (Ca addition), R4 (enzyme addition) and R5 (Ca and enzyme addition)

**Fig. 2:**
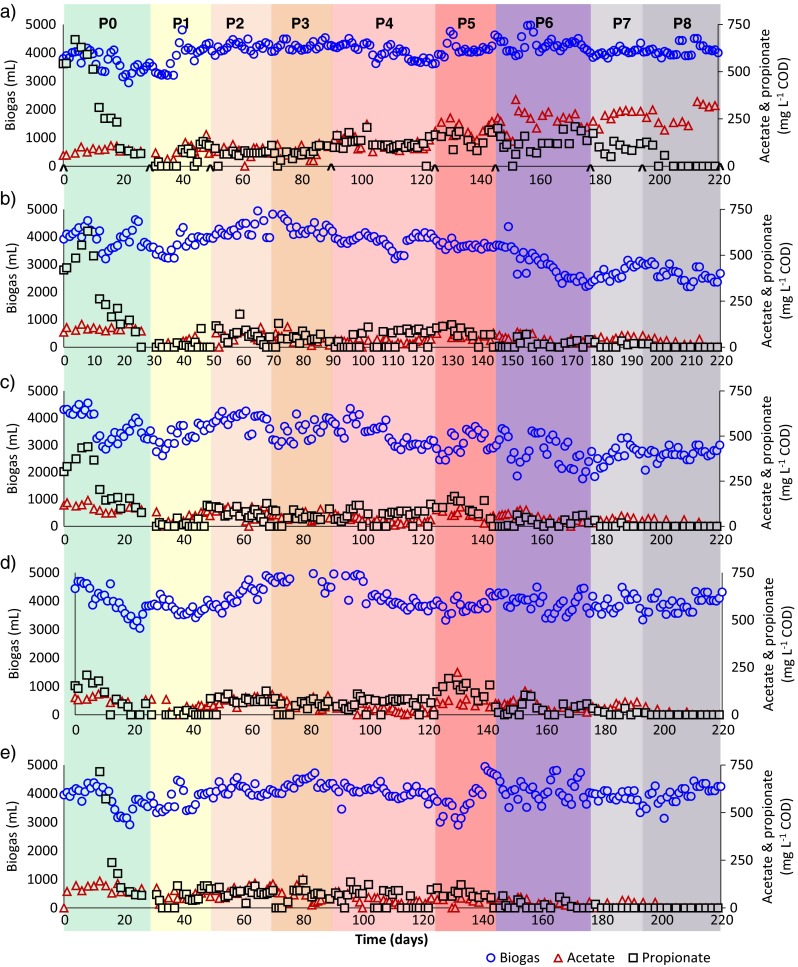
Daily biogas and VFA production of the **a** R1 (control), **b** R2 (inhibition), **c** R3 (Ca addition), **d** R4 (enzyme addition) and **e** R5 (Ca and enzyme addition). *Caret symbol* marks the sampling time points for the microbiological analyses

Figures 1 and 2 and Supplementary Table S1 show the process parameters of each reactor. Until the end of P3, all the reactors followed similar trends in terms of hydrolysis, acidogenesis and methanogenesis efficiencies. During these periods, hydrolysis, acidogenesis and methanogenesis efficiencies of the reactors were calculated to be 51 ± 4%; a stable reactor performance was shown. In all reactors, some acetate and propionate were present and acetate was the dominant VFA. The average total VFA concentration in the reactors was 188 ± 140 mg L^−1^ COD. Measured average daily biogas production in the reactors was 3995 ± 362 mL, and the average methane content of the produced biogas was 51 ± 1% (Fig. 2 and Supplementary Table S1).

Daily addition of HA at concentration as high as 40 mg L^−1^ to the reactors did not show any significant effect till the end of P3. After that period, performance of R2 reduced compared to R1 (blank control). Slow reduction in hydrolysis efficiency was related to increased HA addition. After P3, hydrolysis efficiencies in R2 and R3 decreased gradually from 51 ± 4 to 30 ± 5% throughout the experiment due to the increasing concentration of HA (Fig. 1). Acidogenesis and methanogenesis efficiencies concomitantly decreased due to the restrained hydrolysis. The average total VFA concentration in those reactors remained similar as compared that in the former periods and below 100 mg L^−1^ COD (Fig. 2 and Supplementary Table S1). Average daily biogas production and the methane content of the reactors also reduced after P4. During P7 and P8, measured average daily biogas production in R2 and R3 was 2680 ± 10 mL, which was significantly lower than that in the other reactors (Fig. 2 and Supplementary Table S1). In contrast to R2 and R3, the performance of the other reactors stayed stable throughout the experiment. Hydrolysis efficiencies were calculated to be 53 ± 3% for R1, R4 and R5 after P4. Acidogenesis and methanogenesis efficiencies coincided with hydrolysis efficiencies which showed the process stability of the reactors. VFA concentration in R1 was significantly higher than that in the other reactors, around 350 mg L^−1^ COD from P5 to P8, whereas VFA concentration in R4 and R5 remained low and was not significantly different. In R1, R4 and R5, daily biogas production showed similar trends: 4019 ± 111 mL with a methane content of 50 ± 1% (Supplementary Table S1). Since the enzyme mixtures were partially a source for methane production in R4 and R5, the amount of methane that could be derived from enzymes was subtracted from overall methane production. The methane production from 1 mL enzyme mixture was determined as 70, 80 and 60 mL methane for cellulase, xylanase and endoglucanase, respectively. After subtraction, hydrolysis, acidogenesis and methanogenesis efficiencies were calculated. Thus, the calculated net efficiencies in these reactors were found to be similar to the efficiency of the control reactor (Fig. 1, Supplementary Table S1).

### Bacterial and archaeal community composition

**Fig. 3:**
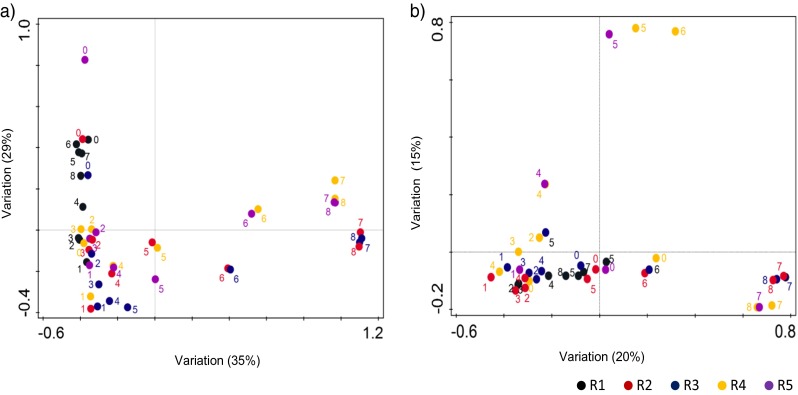
Redundancy analysis (RDA) scatter plots of each individual samples. *Numbers* represent the sampling point of each individual samples. These graphs show the variations between samples according to **a** bacterial community composition and **b** archaeal community composition

The composition of bacterial and archaeal communities plays an important role in anaerobic cellulose and xylan degradation. Addition of HA showed a selective effect on bacterial and archaeal communities. As the HA concentration increased, hydrolysis became restrained and therefore bacterial and archaeal compositions shifted in the reactors R2 to R5. Shifts in the microbial communities occurred after P4 when HA inhibition was observed. Variations in bacterial and archaeal community composition for all reactors in different operational periods are given in Fig. 3. Additionally, rarefaction curves, generated to estimate the coverage of the microbial community in the samples, showed that the plateau phase was reached for all samples, indicating sufficient coverage of the microbial community (Supplementary Fig. S1).

The number of reads per sample for bacterial sequences varied from 1015 to 418,163 (Supplementary Table S2). The reads were assigned to 11 different phyla, 17 classes and 20 orders that were abundant at least 1% of the reads in the samples. The dominant bacterial populations for all reactors affiliated with the phyla *Bacteriodetes*, *Firmicutes* and *Chloroflexi*. In total, 78 ± 12% of the total reads were assigned to those three phyla (Supplementary Fig. S2).

**Fig. 4:**
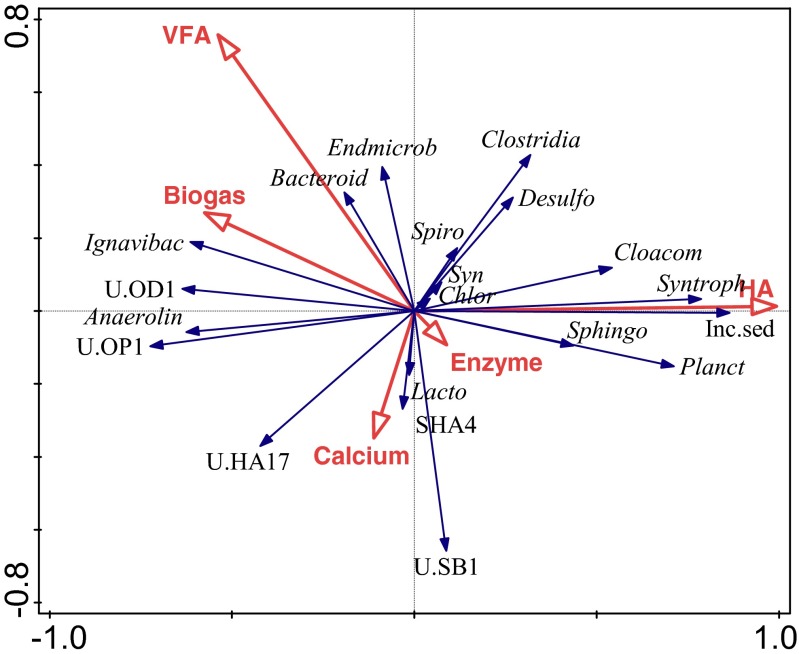
Redundancy analysis (RDA) ordination diplot for the bacterial community. *Red vectors* represent the influence of operational parameters biogas production (*Biogas*), total volatile fatty acids (*Total VFA*), humic acids (*HA*), calcium addition (*Calcium*), enzyme addition (*Enzyme*). *Blue vectors* represent bacterial orders: *Bacteroidales* (*Bacteroid*), Incertae Sedis (*Inc.sed*), Unclassified SB1 (*U.SB1*), *Sphingobacteriales* (*Sphingo*), Unclassified vadin HA17 (*U.HA17*), Unclassified Candidate division OD1 (*U.OD1*), Unclassified Candidate division OP11 (*U.OP1*), *Chlorobiales* (*Chlor*), *Ignavibacteriales* (*Ignavibac*), *Anaerolineales* (*Anaerolin*), Lineage I (*Endmicrob*), *Lactobacillales* (*Lacto*), *Clostridiales* (*Clostridia*), *Planctomycetales* (*Planct*), *Desulfuromonadales* (*Desulfo*), *Syntrophobacterales* (*Syntroph*), *Cloacamonas* (*Cloacom*), SHA4 (*SHA4*), *Spirochaetales* (*Spiro*), *Synergistales* (*Syn*). A detailed correlation matrix is provided in Supplementary Table S3 (colour figure online)

Variations in diversity in lower taxa levels were also observed. In average, 28 ± 11% of the reads could not be assigned at family level, indicating that some of the bacterial populations within the anaerobic sludge remained uncharacterised. At the level of order, *Lactobacillales* (20 ± 12%), *Anaerolineales* (19 ± 9%), *Bacteroidales* (15 ± 9%) and *Clostridiales* (13 ± 7%) were the most abundant within all the reactors throughout the whole experiment (Supplementary Fig. S2). Between these orders, *Bacteroidales* were more dominant in the reactors in which hydrolysis was not inhibited (R1, R4 and R5) than R2–R3 (hydrolysis inhibition). Their relative abundance was associated with biogas production and correlated with VFA concentrations (*r* = 0.372, *p* < 0.01) (Fig. 4, Supplementary Table S3). In the presence of HA, the relative abundance of *Bacteroidales* was reduced by up to 30% (*r* = −0.326, *p* < 0.05) at increasing concentrations of HA in R2 and R3 while their relative abundance within the other reactors stayed relatively stable (Supplementary Fig. S2). *Anaerolineales* was the other abundant bacterial order within all reactors. *Anaerolineales* significantly correlated with biogas production (*r* = 0.477, *p* < 0.001) (Supplementary Table S3). *Anaerolineales* was negatively affected (*r* = −0.355, *p* < 0.05) by the increasing concentrations of HA (Supplementary Table S3). In R2 and R3 (hydrolysis inhibition), the relative abundance of *Anaerolineales* was reduced threefold as compared to the other reactors (Supplementary Fig. S2). On the other hand, not many bacterial groups correlated with the presence of calcium and enzyme addition. *Lactobacillales*, *Spirochaetes-*SHA-4 and Unclassified *Bacteriodetes* class SB-1 were mainly clustered with enzyme and calcium addition (Fig. 4).

The number of reads per sample for archaeal sequences varied from 1029 to 170,459 (Supplementary Table S2). The samples (R1-P6, R5-P3, P6 and P8) that had lower than 1000 reads were not included in the statistical analyses, but they were represented in Supplementary Fig. S3. All reads were assigned to phylum *Euryarchaeota*, within four different classes, six orders and nine families abundant in at least 1% of the reads in the samples. Of the reads, 4 ± 3% could not be assigned at family level. The archaeal community structure was more stable than the bacterial community (Fig. 3b). The dominant archaeal population in all reactors at the family level were members of *Methanospirillaceae* (37 ± 21%), *Methanobacteriaceae* (27 ± 19%), *Methanoregulaceae* (10 ± 14%) and *Methanosaetaceae* (10 ± 8%). On average, 81 ± 11% of the reads affiliated with those four families in all the reactors. Except *Methanosaetaceae* and *Methanosarcinaceae*, which have members that perform acetoclastic methanogenesis, the other family groups included hydrogenotrophic methanogens. Beside the aforementioned families, members of the order *Methanosarcinales*, *Methanomicrobiales* and *Thermoplasmatales* were also detected at low levels (<5%) within the reactors in various relative abundance (Supplementary Fig. S3).

**Fig. 5:**
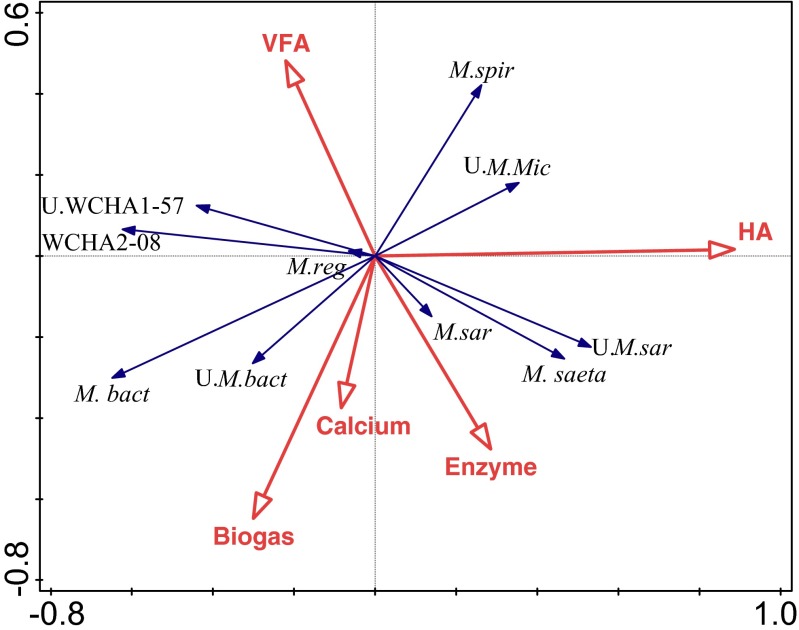
Redundancy analysis (RDA) ordination diplot for the archaeal community. *Red vectors* represent the influence of operational parameters biogas production (*Biogas*), total volatile fatty acids (*Total VFA*), humic acids (*HA*), calcium addition (*Calcium*), enzyme addition (*Enzyme*). *Blue vectors* represent archaeal families: *Methanobacteriaceae* (*M. bact*), Unassigned *Methanobacteriales* (U.*M. bact*), *Methanoregulaceae* (*M.reg*), *Methanospirillaceae* (*M. spir*), *Methanomicrobiales* WCHA2-08 (WCHA2-08), Unassigned *Methanomicrobiales* (U.*M. Mic*), *Methanosaetaceae* (*M.saeta*), *Methanosarcinaceae* (*M.sar*), Unassigned *Methanosarcinales* (U.*M.sar*), Unassigned *Thermoplasmata* WCHA-1-57 (U. WCHA1-57). A detailed correlation matrix provided as Supplementary Table S4

Members of *Methanobacteriaceae* and *Methanoregulaceae* were found related with biogas production (Fig. 5). Especially *Methanobacteriaceae* were significantly (*r* = 0.300, *p* < 0.05) correlated with biogas production (Supplementary Table S4). The presence of HA affected the archaeal composition. The relative abundance of *Methanobacteriaceae*, *Methanomicrobiales-*WCHA208 and Unassigned *Thermoplasmata-*WCHA1-57 were negatively affected by the presence of HA. Their relative abundance showed significant negative correlation (*r* = −0.400, *p* < 0.01) with the presence of HA. On the other hand, members of the acetoclastic methanogenic family *Methanosaetaceae* showed significant positive correlation (*r* = 0.589, *p* < 0.001) with the presence of HA (Supplementary Table S4). This result suggested that the relative abundance of *Methanosaetaceae* increased while the relative abundance of hydrogenotrophic methanogens decreased in R2–R5.

Calcium and enzyme addition were not deterministic for archaeal composition (Fig. 5). *Methanobacteriaceae* were positively correlated with elevated calcium concentrations whereas, *Methanospirillaceae* (*r* = −0.340, *p* < 0.05) and Unclassified *Methanomicrobiales* (*r* = −0.350, *p* < 0.01) were found negatively correlated (Supplementary Table S4). There was only one family showing a negative correlation with enzyme additions, which was *Methanospirillaceae* (*r* = −0.302, *p* < 0.05) (Supplementary Table S4).

## Discussion

### Effect of HA addition on digester performance

In this research, the effect of HA on the anaerobic digestion of xylan and cellulose was evaluated by calculating hydrolysis, acidogenesis and methanogenesis efficiencies. Hydrolysis efficiency of R2 was reduced by 40%, compared to the control reactor when the HA concentrations reached up to around 8 g L^−1^. This confirms the previous observations of HA inhibition in batch incubations (Fernandes et al. 2015; Azman et al. 2015b). Restrained hydrolysis in R2 influenced the subsequent steps of the anaerobic digestion, causing reduced biogas production after P3. However, the degree of inhibition was not similar between the reported inhibition levels. Fernandes et al. (2015) extracted HA from maize and manure and reported inhibitory concentrations of HA on batch-wise enzymatic cellulose degradation as low as 0.5 g L^−1^, whereas Azman et al. (2015b) reported 50% inhibition on anaerobic degradation of cellulose by using commercially available humic acid salts at 5 g L^−1^ concentrations in batch incubations. As can be understood from previous studies, when comparing the reported inhibition values, it is not possible to define a specific HA inhibition value for anaerobic digestion. This is mainly related to the composition and dosing strategies of the HA used in the studies. HA are complex molecules, their compositions vary drastically with the source of HA, extraction and preparation methods (Tan 2014). The effects of HA seem to be case specific and should be evaluated separately for each bioreactor and used feedstock. In this study, we observed hydrolysis inhibition around 8 g L^−1^ by using the same stock of HA that was used previously (Azman et al. 2015). The observed inhibition concentration in our study are much higher than the reported HA concentrations from plant material and manure (Fernandes 2010). The main reason for the differences in the observed inhibition levels can be related to adaptation capacity of the microbial community to elevated HA concentrations. Step-wise increase of the HA that might enable the microbial communities to adapt to the concentrations can be attributed to the acclimation capacity of the microbial communities to HA. Potential acclimation of microorganisms can be possible either via production of more hydrolytic enzymes as Fernandes et al. (2015) suggested or production of extra polymeric substance (EPS)-like molecules by different microbial communities to prevent HA from penetrating the active cells and disrupt the cell integrity (Prokhotskaya and Steinberg 2007). Additionally, aforementioned studies mainly reported acute effects of HA. Because of that, HA inhibition may be different in continuous reactor systems when compared to batch systems and show more chronic effects.

### Mitigation of HA inhibition by calcium and hydrolytic enzyme addition

CaCl_2_ was added daily to R3 and R5 to evaluate the potential of calcium to mitigate HA inhibition. Positive effects of calcium addition to overcome HA inhibition were reported previously in batch-wise incubations. Brons et al. (1985) reported the mitigation of HA inhibition on potato starch hydrolysis via CaCl_2_ addition and Azman et al. (2015b) observed similar effects on cellulose hydrolysis. In general, calcium is considered an essential macronutrient to support microbial growth and aggregate formation. Addition of CaCl_2_ is known to have a stimulatory effect on anaerobic digestion in the range of 100 to 3000 mg L^−1^ (Chen et al. 2008; Romero-Güiza et al. 2016). However, our study showed different results compared to previous studies. In R3, in which 0.11 g CaCl_2_ g HA_added_^−1^ was added as mitigation agent for HA inhibition, hydrolysis efficiencies remained similar to the reactor in which HA were added daily without CaCl_2_ addition (R2). Our results might be explained by restrained surface availability of cellulose and xylan particles due to the formation of HA-calcium precipitates (Alvarez et al. 2004). Precipitates that accumulated in the completely stirred tank reactor (CSTR) could have prevented adhesion of the microorganisms to the cellulose and xylan particles which were crucial for hydrolytic activity. Another reason could be related with the calcium addition strategy. Azman et al. (2015b) used pulse addition of 5 mM CaCl_2_, whereas in this study, semi-continuous addition of CaCl_2_ was applied. Pulse addition of 2.5 mM CaCl_2_ at short HRTs (2–4 h) was shown to even enhance anaerobic digestion of sucrose by a mixed culture (Yuan et al. 2010). Therefore, the observations of Azman et al. (2015b) might be more related to enhancement of microbial activity, leading to more enzyme production rather than mitigation of the HA inhibition. Difference in calcium addition strategies between batch-wise incubations and continuous operation could also cause different results which suggested to study further to find the optimum calcium dosing strategies for anaerobic digestion.

On the other hand, enzyme addition to R4 and R5 showed a positive effect to overcome HA addition. The positive results indicated that the effects of HA were reversed by enzyme addition. Two hypotheses can be postulated to explain the positive effect of the enzyme addition: (i) additional hydrolytic enzymes can attach to humic acids, preventing their scavenging behaviour against intrinsic hydrolytic enzyme production by abundant hydrolytic bacteria within anaerobic sludge (Fernandes et al. 2015); (ii) competition between HA and enzymes to bind the cellulose particles. Lignin has similar functional groups as HA, and Vermaas et al. (2015) found that lignin preferentially binds to the hydrophobic side of the cellulose and also to the specific residues on the cellulose-binding modules of the enzymes that are critical for cellulose binding to cellulases. Our results can support both hypotheses by showing that the intrinsic enzyme production from hydrolytic bacteria can maintain the hydrolytic activity when binding sites of HA were inactivated by enzyme addition or preventing HA to bind cellulose particles.

Interestingly, when enzyme and calcium were added together, the same effect was observed as with the sole enzyme addition. For R3, we hypothesised that precipitates of the HA-Ca complex might cover cellulose and xylan particles, preventing enzyme adhesion and consequently lower the biodegradation. In contrast to R3, in R5, enzyme-humic acid binding might be stronger than enzyme-calcium bindings and affinity of enzymes to HA might be higher than to calcium. It is known that hydrolytic enzymes (especially β-glucosidases) form very strong bonds in soil environments (Ceccanti et al. 2008). Once active binding sites of the HA are occupied by hydrolytic enzymes, it is more likely that calcium-HA complexes are not formed, and consequently, calcium cations can enhance anaerobic digestion as discussed previously (Yuan et al. 2010; Romero-Güiza et al. 2016). However, this hypothesis needs further studies to be proven.

### Effect of HA, enzyme and calcium addition on bacterial and archaeal community composition

Microbiological analyses showed that members of *Bacteriodetes* and *Firmicutes* were present in all reactors, suggesting that they shaped the core bacterial population involved in anaerobic cellulose and xylan degradation. *Bacteriodetes* and *Firmicutes* are well-known fermentative hydrolytic bacteria that are responsible for anaerobic cellulose degradation in many biogas plants (Azman et al. 2015a; Campanaro et al.^2016^; De Vrieze et al. 2015a; Stolze et al. 2015; Westerholm et al. 2016). Additionally, hydrolytic enzyme production of *Bacteriodetes* and *Firmicutes* has been shown previously in both reactor and gut environments (Azman et al. 2015a; Hong et al. 2014; Zhang et al. 2014). In all reactors, higher relative abundance of *Bacteroidales* and *Clostridiales* indicated that these were the key players in the hydrolysis of cellulose and xylan. They are important for xylan and cellulose degradation, forming intermediate products such as short-chain fatty acids and H_2_. In the presence of HA, the relative abundance of *Clostridiales* was correlated with the presence of HA than *Bacteroidales*, suggesting that ongoing hydrolysis in R4 and R5 might be maintained by members of this order (Fig. 5). Members of *Anaerolineales* are known to ferment sugars in anaerobic digesters (Ambuchi et al. 2016; de Vrieze et al. 2015b), and they have a role in degradation of a variety of carbohydrates, including xylan (Yamada et al. 2007). The high frequency and dominant co-occurrence of *Anaerolineales* with cellulolytic species indicated the possible interaction between them during cellulose and xylan hydrolysis. There are no many cultured *Anaerolineales* species, but cultured *Anaerolineales* species grow together with a hydrogenotrophic partner (Sekiguchi et al. 2001; Yamada et al. 2006). Because of that reason, growth of *Anaerolineales* could be dependent on hydrogenotrophic methanogens. Decrease in the relative abundance of *Anaerolineales* suggests the disruption in their possible microbial interactions with methanogens, leading to decreased methane production. When the relative abundance of *Anaerolineales* reduced due to the presence of HA, a well-known syntrophic group *Syntrophobacterales* increased in abundance in the R2 to R5. Their relative abundance was correlated with the HA (*r* = 0.569, *p* < 0.001). Interestingly, we did not detect *Syntrophobacterales* in the control reactors. Most probably, they could not compete with the *Anaerolineales* species. *Planctomycetales* was the other bacterial order that was positively correlated with HA (*r* = 0.584, *p* < 0.001). Members of *Planctomycetales* are highly diverse, and their role in nature is mostly unclear. Some members are thought to be involved in humus degradation in termite gut (Kudo 2009; Ward et al. 2006). Therefore, their relative abundance in R2 to R5 might be related to HA degradation. However, more research is required to test this occurrence.

In all reactors, *Lactobacillales* was represented by only *Trichococcus* genus. *Trichococcus* species can be frequently found in wastewater treatment plants. Especially, *Trichococcus flocculiformis* was reported to cause foaming and bulking of the sludge which is not desirable for reactor operations (Nielsen et al. 2009; Scheff et al. 1984). We did observe foaming in R3–R5 whereas no foaming in R1 and moderate foaming in R2 was observed. The relative abundance of *Trichococcus* in these reactors most probably caused the foaming problem. Though foaming problems were prevented with the addition of equal amounts of silicone oil to all reactors, the relative abundance of *Trichococcus* was not reduced in R2–R5.

In addition to bacterial communities, the methanogenic communities were also affected by the reactor conditions. The relative abundance of *Methanobacteriaceae* with positive correlation with biogas production was reported previously in high rate AD systems (de Vrieze et al. 2015b; Hao et al. 2012; Steinberg and Regan 2011). In the absence of HA, *Methanoregulaceae* became relatively dominant at the end of the experiment in R1. The members of *Methanoregulaceae* use H_2_/CO_2_ and also formate (Oren 2014). They have been reported in relatively low amounts (relative abundance 1–15%) in anaerobic digesters (Vanwonterghem et al. 2015; Wilkins et al. 2015). In our study, their relative abundance increased up to 35%. It was not clear why *Methanoregulaceae* became highly abundant in R1.

The presence of HA negatively affected the relative abundance of hydrogenotrophic methanogens. Decreased relative abundance of *Methanomicrobiales-*WCHA208 and Unassigned *Thermoplasmata* WCHA1-57 possibly resulted with decreased level of hydrogenotrophic methanogenesis. On the other hand, the increase in the relative abundance of *Methanosaetaceae* can be attributed to their acclimation capacity to increasing HA concentrations. Low acetate concentrations in HA-containing reactors, compared to the control reactor, may also support the role of *Methanosaetaceae* in these reactors. Their high affinity of acetate in mesophilic conditions has been demonstrated previously (Conklin et al. 2006).

In conclusion, HA inhibited especially the hydrolysis step of the digestion up to 40%. Addition of hydrolytic enzymes helped to reverse the negative effects of HA whereas calcium addition did not show any effects to reverse HA inhibition. Microbiological analyses showed that fermentative hydrolytic bacteria and hydrogenotrophic methanogens were affected by the presence of HA, whereas acetoclastic methanogens were not affected by HA addition. Our results showed that intrinsic enzyme production was sufficient to maintain hydrolytic activity when there were no active enzyme scavengers in the environment. For that reason, we propose to control enzyme additions based on the influent HA rather than volatile solid concentration, to limit costs.

## Electronic supplementary material

ESM 1 (PDF 864 kb).

## References

[CR1] Alvarez R, Evans LA, Milham PJ, Wilson MA (2004). Effects of humic material on the precipitation of calcium phosphate. Geoderma.

[CR2] Ambuchi JJ, Liu J, Wang H, Shan L, Zhou X, Mohammed MO, Feng Y (2016). Microbial community structural analysis of an expanded granular sludge bed (EGSB) reactor for beet sugar industrial wastewater (BSIW) treatment. Appl Microbiol Biotechnol.

[CR3] Appels L, Lauwers J, Degrève J, Helsen L, Lievens B, Willems K, Van Impe J, Dewil R (2011). Anaerobic digestion in global bio-energy production: potential and research challenges. Renew Sustainable Energy Rev.

[CR4] Azman S, Khadem AF, van Lier JB, Zeeman G, Plugge CM (2015). Presence and role of anaerobic hydrolytic microbes in conversion of lignocellulosic biomass for biogas production. Crit Rev Env Sci Tec.

[CR5] Azman S, Khadem AF, Zeeman G, van Lier JB, Plugge CM (2015). Mitigation of humic acid inhibition in anaerobic digestion of cellulose by addition of various salts. Bioengineering.

[CR6] Brons HJ, Field JA, Lexmond WAC, Lettinga G (1985). Influence of humic acids on the hydrolysis of potato protein during anaerobic digestion. Agricultural Wastes.

[CR7] Campanaro S, Treu L, Kougias PG, Francisci D, Valle G, Angelidaki I (2016). Metagenomic analysis and functional characterization of the biogas microbiome using high throughput shotgun sequencing and a novel binning strategy. Biotechnol Biofuels.

[CR8] Caporaso JG, Kuczynski J, Stombaugh J, Bittinger K, Bushman FD, Costello EK, Fierer N, Peña AG, Goodrich JK, Gordon JI, Huttley GA, Kelley ST, Knights D, Koenig JE, Ley RE, Lozupone CA, McDonald D, Muegge BD, Pirrung M, Reeder J, Sevinsky JR, Turnbaugh PJ, Walters WA, Widmann J, Yatsunenko T, Zaneveld J, Knight R (2010). QIIME allows analysis of high-throughput community sequencing data. Nat Methods.

[CR9] Ceccanti B, Doni S, Macci C, Cercignani G, Masciandaro G (2008). Characterization of stable humic–enzyme complexes of different soil ecosystems through analytical isoelectric focussing technique (IEF). Soil Biol Biochem.

[CR10] Chen Y, Cheng JJ, Creamer KS (2008). Inhibition of anaerobic digestion process: a review. Bioresour Technol.

[CR11] Conklin A, Stensel HD, Ferguson J (2006). Growth kinetics and competition between *Methanosarcina* and *Methanosaeta* in mesophilic anaerobic digestion. Water Environ Res.

[CR12] Daims H, Brühl A, Amann R, Schleifer KH, Wagner M (1999). The domain-specific probe EUB338 is insufficient for the detection of all bacteria: development and evaluation of a more comprehensive probe set. Syst Appl Microbiol.

[CR13] Davies G, Ghabbour EA, Steelink C (2001) Humic acids: marvelous products of soil chemistry. J Chem Educ 78:1609–1614. doi:10.1021/ed078p1609

[CR14] De Vrieze J, Gildemyn S, Vilchez-Vargas R, Jáuregui R, Pieper DH, Verstraete W, Boon N (2015a) Inoculum selection is crucial to ensure operational stability in anaerobic digestion. Appl Microbiol Biotechnol 99:189–199. doi:10.1007/s00253-014-6046-3 10.1007/s00253-014-6046-3 25261127

[CR15] De Vrieze J, Saunders AM, He Y, Fang J, Nielsen PH, Verstraete W, Boon N (2015). Ammonia and temperature determine potential clustering in the anaerobic digestion microbiome. Water Res.

[CR16] El-Mashad HM, Zeeman G, Van Loon WK, Bot GP, Lettinga G (2004). Effect of temperature and temperature fluctuation on thermophilic anaerobic digestion of cattle manure. Bioresour Technol.

[CR17] Fernandes TV (2010) Hydrolysis inhibition of complex biowaste. Dissertation, Wageningen University

[CR18] Fernandes TV, van Lier JB, Zeeman G (2015). Humic acid-like and fulvic acid-like inhibition on the hydrolysis of cellulose and tributyrin. Bioenergy Res.

[CR19] Hamady M, Walker JJ, Harris JK, Gold NJ, Knight R (2008). Error-correcting barcoded primers allow hundreds of samples to be pyrosequenced in multiplex. Nat Methods.

[CR20] Hao LP, Lü F, Li L, Shao LM, He PJ (2012). Shift of pathways during initiation of thermophilic methanogenesis at different initial pH. Bioresour Technol.

[CR21] Hendriks ATWM, Zeeman G (2009). Pretreatments to enhance the digestibility of lignocellulosic biomass. Bioresour Technol.

[CR22] Hong P-Y, Iakiviak M, Dodd D, Zhang M, Mackie RI, Cann I (2014). Two new xylanases with different substrate specificities from the human gut bacterium *Bacteroides intestinalis* DSM 17393. Appl Environ Microbiol.

[CR23] Kang K-H, Shin HS, Park H (2002). Characterization of humic substances present in landfill leachates with different landfill ages and its implications. Water Res.

[CR24] Klinke HB, Thomsen AB, Ahring BK (2004). Inhibition of ethanol-producing yeast and bacteria by degradation products produced during pre-treatment of biomass. Appl Microbiol Biotechnol.

[CR25] Kopetz H (2013). Renewable resources: build a biomass energy market. Nature.

[CR26] Kudo T (2009). Termite-microbe symbiotic system and its efficient degradation of lignocellulose. Biosci Biotechnol Biochem.

[CR27] Kvist T, Ahring BK, Westermann P (2007). Archaeal diversity in Icelandic hot springs. FEMS Microbiol Ecol.

[CR28] Lauri P, Havlík P, Kindermann G, Forsell N, Böttcher H, Obersteiner M (2014). Woody biomass energy potential in 2050. Energy Policy.

[CR29] Li X, Xing M, Yang J, Huang Z (2011). Compositional and functional features of humic acid-like fractions from vermicomposting of sewage sludge and cow dung. J Hazard Mater.

[CR30] Li H, Li Y, Jin Y, Zou S, Li C (2014). Recovery of sludge humic acids with alkaline pretreatment and its impact on subsequent anaerobic digestion. J Chem Technol Biotechnol.

[CR31] Liu X, Bayard R, Benbelkacem H, Buffière P, Gourdon R (2015). Evaluation of the correlations between biodegradability of lignocellulosic feedstocks in anaerobic digestion process and their biochemical characteristics. Biomass Bioenerg.

[CR32] Negro MJ, Manzanares P, Oliva JM, Ballesteros I, Ballesteros M (2003). Changes in various physical/chemical parameters of *Pinus pinaster* wood after steam explosion pretreatment. Biomass Bioenerg.

[CR33] Nielsen PH, Kragelund C, Seviour RJ, Nielsen JL (2009). Identity and ecophysiology of filamentous bacteria in activated sludge. FEMS Microbiol Rev.

[CR34] Oren A, Rosenberg E, DeLong EF, Lory S, Stackebrandt E, Thompson F (2014). The family *Methanoregulaceae*. The prokaryotes.

[CR35] Plugge CM (2005). Anoxic media design, preparation, and considerations. Methods Enzymol.

[CR36] Prokhotskaya VY, Steinberg CE (2007). Differential sensitivity of a coccal green algal and a cyanobacterial species to dissolved natural organic matter (NOM). Environ Sci Poll Res.

[CR37] Quast C, Pruesse E, Yilmaz P, Gerken J, Schweer T, Yarza P, Peplies J, Glöckner FO (2013). The SILVA ribosomal RNA gene database project: improved data processing and web-based tools. Nucleic Acids Res.

[CR38] Ramiro-Garcia J, Hermes GDA, Giatsis C, Sipkema D, Zoetendal EG, Schaap PJ, Smidt H (2016). NG-tax, a highly accurate and validated pipeline for analysis of 16S rRNA amplicons from complex biomes [version 1; referees: awaiting peer review]. F1000Research.

[CR39] Raposo F, De la Rubia M, Fernández-Cegrí V, Borja R (2012). Anaerobic digestion of solid organic substrates in batch mode: an overview relating to methane yields and experimental procedures. Renew Sustainable Energy Rev.

[CR40] Ren Z, Graham N (2015). Treatment of humic acid in drinking water by combining potassium manganate (Mn (Vi)), ferrous sulfate, and magnetic ion exchange. Environ Eng Sci.

[CR41] Romero-Güiza MS, Vila J, Mata-Alvarez J, Chimenos JM, Astals S (2016). The role of additives on anaerobic digestion: a review. Renew Sustainable Energy Rev.

[CR42] Sawin JL, Sverrisson F, Rickerson W, Lins C, Williamson LE, Adib R, Murdock HE, Musolino E, Hullin M, Reith A, Valero A, Mastny L, Petrichenko K, Seyboth K, Skeen J, Sovacool B, Wouters F, Martinot E (2015). Renewables 2015 global status report- annual reporting on renewables: ten years of excellence (INIS-FR--15-0643).

[CR43] Scheff G, Salcher O, Lingens F (1984). *Trichococcus flocculiformis* gen. nov. sp. nov. A new gram-positive filamentous bacterium isolated from bulking sludge. Appl Microbiol Biotechnol.

[CR44] Sekiguchi Y, Takahashi H, Kamagata Y, Ohashi A, Harada H (2001). In situ detection, isolation, and physiological properties of a thin filamentous microorganism abundant in methanogenic granular sludges: a novel isolate affiliated with a clone cluster, the green non-sulfur bacteria, subdivision I. Appl Environ Microbiol.

[CR45] Šmilauer P, Lepš J (2014). Multivariate analysis of ecological data using CANOCO 5.

[CR46] Song H, Li A, Zhou Y, Xu J, Wu J, He Y (2013). Selective removal of DOM on anion-exchange resin from water. Functions of natural organic matter in changing environment.

[CR47] Stams AJM, Van Dijk JB, Dijkema C, Plugge CM (1993). Growth of syntrophic propionate-oxidizing bacteria with fumarate in the absence of methanogenic bacteria. Appl Environ Microbiol.

[CR48] Steinberg LM, Regan JM (2011). Response of lab-scale methanogenic reactors inoculated from different sources to organic loading rate shocks. Bioresour Technol.

[CR49] Stolze Y, Zakrzewski M, Maus I, Eikmeyer F, Jaenicke S, Rottmann N, Siebner C, Pühler A, Schlüter A (2015). Comparative metagenomics of biogas-producing microbial communities from production-scale biogas plants operating under wet or dry fermentation conditions. Biotechnol Biofuels.

[CR50] Tan KH (2014). Humic matter in soil and the environment: principles and controversies.

[CR51] Tiwary A, Williams ID, Pant DC, Kishore VVN (2015). Emerging perspectives on environmental burden minimisation initiatives from anaerobic digestion technologies for community scale biomass valorisation. Renew Sustainable Energ Rev.

[CR52] Toka A, Iakovou E, Vlachos D, Tsolakis N, Grigoriadou A-L (2014). Managing the diffusion of biomass in the residential energy sector: an illustrative real-world case study. Appl Energy.

[CR53] van den Bogert B, de Vos WM, Zoetendal EG, Kleerebezem M (2011). Microarray analysis and barcoded pyrosequencing provide consistent microbial profiles depending on the source of human intestinal samples. Appl Environ Microbiol.

[CR54] van den Bogert B, Erkus O, Boekhorst J, de Goffau M, Smid EJ, Zoetendal EG, Kleerebezem M (2013). Diversity of human small intestinal *Streptococcus* and *Veillonella* populations. FEMS Microbiol Ecol.

[CR55] van Meerbeek K, Appels L, Dewil R, van Beek J, Bellings L, Liebert K, Muys B, Hermy M (2015). Energy potential for combustion and anaerobic digestion of biomass from low-input high-diversity systems in conservation areas. GCB Bioenergy.

[CR56] Vanwonterghem I, Jensen PD, Rabaey K, Tyson GW (2015). Temperature and solids retention time control microbial population dynamics and volatile fatty acid production in replicated anaerobic digesters. Sci Rep.

[CR57] Vassilev SV, Baxter D, Andersen LK, Vassileva CG (2010). An overview of the chemical composition of biomass. Fuel.

[CR58] Vermaas JV, Petridis L, Qi X, Schulz R, Lindner B, Smith JC (2015). Mechanism of lignin inhibition of enzymatic biomass deconstruction. Biotechnol Biofuels.

[CR59] Wang Y, Qian P-Y (2009). Conservative fragments in bacterial 16S rRNA genes and primer design for 16S ribosomal DNA amplicons in metagenomic studies. PLoS One.

[CR60] Ward N, Staley JT, Fuerst JA, Giovannoni S, Schlesner H, Stackebrandt E, Dworkin M, Falkow S, Rosenberg E, Schleifer K-H, Stackebrandt E (2006). The order *Planctomycetales*, including the genera *Planctomyces*, *Pirellula*, *Gemmata* and *Isosphaera* and the candidatus genera *Brocadia*, *Kuenenia* and *Scalindua*. The prokaryotes.

[CR61] Westerholm M, Crauwels S, van Geel M, Dewil R, Lievens B, Appels L (2016). Microwave and ultrasound pre-treatments influence microbial community structure and digester performance in anaerobic digestion of waste activated sludge. Appl Microbiol Biotechnol.

[CR62] Wilkins D, Rao S, Lu X, Lee PKH (2015). Effects of sludge inoculum and organic feedstock on active microbial communities and methane yield during anaerobic digestion. Front Microbiol.

[CR63] Yamada T, Sekiguchi Y, Hanada S, Imachi H, Ohashi A, Harada H, Kamagata Y (2006). *Anaerolinea thermolimosa* sp. nov., *Levilinea saccharolytica* gen. nov., sp. nov. and *Leptolinea tardivitalis* gen. nov., sp. nov., novel filamentous anaerobes, and description of the new classes *Anaerolineae* classis nov. and *Caldilineae* classis nov. in the bacterial phylum *Chloroflexi*. Int J Syst Evol Microbiol.

[CR64] Yamada T, Imachi H, Ohashi A, Harada H, Hanada S, Kamagata Y, Sekiguchi Y (2007). *Bellilinea caldifistulae* gen. nov., sp. nov. and *Longilinea arvoryzae* gen. nov., sp. nov., strictly anaerobic, filamentous bacteria of the phylum *Chloroflexi* isolated from methanogenic propionate-degrading consortia. Int J Syst Evol Microbiol.

[CR65] Yuan Z, Yang H, Zhi X, Shen J (2010). Increased performance of continuous stirred tank reactor with calcium supplementation. Int J Hydrog Energy.

[CR66] Zhang M, Chekan JR, Dodd D, Hong P-Y, Radlinski L, Revindran V, Nair SK, Mackie RI, Cann I (2014). Xylan utilization in human gut commensal bacteria is orchestrated by unique modular organization of polysaccharide-degrading enzymes. Proc Natl Acad Sci.

[CR67] Zheng Y, Zhao J, Xu F, Li Y (2014). Pretreatment of lignocellulosic biomass for enhanced biogas production. Prog Energy Combust Sci.

