# Supplementary material for: Effect of humic acid on anaerobic digestion of cellulose and xylan in completely stirred tank reactors: inhibitory effect, mitigation of the inhibition and the dynamics of the microbial communities
Source: Appl Microbiol Biotechnol. 2016 Nov 29;101(2):889–901. doi: 10.1007/s00253-016-8010-x (PMC5219019; doi:10.1007/s00253-016-8010-x)
Supplement: Supplementary file 1 — (PDF 864 kb). [file 253_2016_8010_MOESM1_ESM.pdf]

Applied Microbiology and Biotechnology

Effect of humic acid on anaerobic digestion of cellulose and xylan in completely stirred tank reactors: inhibitory effect, mitigation of the inhibition and the dynamics of the microbial communities.

Samet Azman [a, c *], Ahmad F. Khadem[a, b], Caroline M. Plugge[a], Alfons J.M. Stams[a], Sabina Bec[a] and Grietje Zeeman[c]

[a] Laboratory of Microbiology, Wageningen University, Stippeneng 4, 6708 WE Wageningen, The Netherlands.

[b] Faculty of Civil Engineering and Geosciences, Department of Water Management, Section Sanitary Engineering, Delft University of Technology, Stevinweg 1, 2628 CN Delft, The Netherlands.

[c] Sub-department of Environmental Technology, Wageningen University, Bornse Weilanden 9, 6708 WG Wageningen, The Netherlands.

*Corresponding Author: Samet Azman samet.azman@wur.nl; Tel: +31 317 483116; Fax: +31 317 483829

Supplementary Table S1 Summary of reactor operation data within different time periods

Reactor	Parameter	Unit	P0		P1		P2		P3		P4		P5		P6		P7		P8	
			x̄	σ	x̄	σ	x̄	σ	x̄	σ	x̄	σ	x̄	σ	x̄	σ	x̄	σ	x̄	σ
R1	Acetate Concentration	mg /L COD	82	16	75	42	76	24	75	34	122	36	203	38	244	56	251	113	270	48
	Propionate Concentration	mg /L COD	346	219	52	49	70	21	70	29	119	35	149	40	116	51	36	42	34	51
	Biogas Production	mL	3677	322	3826	469	4197	153	4243	139	4001	260	4187	246	4309	265	4029	117	4098	189
	Methane Content	%	52.0	1.0	51.0	0.0	51.0	0.0	50.5	0.5	50.0	0.0	50.0	0.0	50.0	0.5	50.0	0.0	50.0	0.0
	Hydrolysis Efficiency	%	50.5	5.5	52.7	1.0	52.0	1.0	54.2	0.5	50.5	2.5	56.0	0.0	58.0	0.0	53.0	0.0	54.0	0.0
	Acidogenesis Efficiency	%	52.3	6.0	50.0	6.6	54.5	2.0	53.7	1.3	51.9	3.4	55.6	3.5	56.3	3.4	53.4	1.7	54.1	2.6
	Methanogenesis Efficiency	%	48.3	4.3	48.5	5.9	53.2	1.9	52.8	1.7	49.7	3.2	52.0	3.1	56.4	3.6	50.1	1.5	50.9	2.3
R2	Acetate Concentration	mg /L COD	100	12	15	19	62	40	46	25	28	16	61	21	43	23	45	13	26	0
	Propionate Concentration	mg /L COD	303	189	12	25	64	43	48	31	45	44	64	41	18	21	13	15	0	0
	Biogas Production	mL	3974	349	3706	318	4307	324	4362	324	3838	246	3672	89	3016	488	2615	259	2669	272
	Methane Content	%	52.5	0.5	52.0	0.0	52.0	0.0	51.5	0.5	50.5	0.5	50.0	0.0	49.0	2.0	45.5	0.5	49.0	0.0
	Hydrolysis Efficiency	%	49.3	0.4	50.0	2.2	54.2	0.4	58.6	3.2	51.1	0.6	45.7	0.0	27.8	0.0	33.2	0.0	30.4	0.0
	Acidogenesis Efficiency	%	55.9	6.0	48.3	4.1	57.1	4.5	56.4	5.2	49.2	3.1	46.9	1.6	38.9	7.5	29.9	2.6	32.6	3.4
	Methanogenesis Efficiency	%	52.3	4.7	47.9	4.1	55.6	4.2	55.4	4.4	48.6	3.1	45.6	1.1	38.9	7.3	29.6	2.7	32.5	3.3
R3	Acetate Concentration	mg /L COD	103	20	46	25	76	31	57	18	33	18	61	20	50	25	35	12	17	18
	Propionate Concentration	mg /L COD	239	131	23	37	71	31	50	27	61	38	93	55	21	21	13	20	0	0
	Biogas Production	mL	3710	500	3330	346	3912	288	3554	347	3366	405	3094	371	2877	518	2560	421	2691	205
	Methane Content	%	52	1	52	0.5	52	0	51.5	0.5	50.0	1.0	49.0	0.0	50.0	1.0	49.0	0.0	50.0	0.0
	Hydrolysis Efficiency	%	46.0	1.0	44.8	4.3	48.8	3.2	46.1	5.8	45.5	7.2	36.0	0.0	32.1	0.0	35.6	0.0	31.6	0.0
	Acidogenesis Efficiency	%	51.9	8.1	42.8	4.7	51.4	4.0	45.8	4.4	43.6	5.4	39.8	3.9	36.4	6.9	32.0	6.1	33.0	1.7
	Methanogenesis Efficiency	%	48.5	6.6	42.2	4.4	50.5	3.7	45.1	4.3	42.6	5.1	37.7	4.5	37.5	6.9	31.4	5.1	32.8	2.4
R4	Acetate Concentration	mg /L COD	72	32	58	24	80	16	58	22	28	18	75	46	54	32	44	17	8	14
	Propionate Concentration	mg /L COD	86	72	93	29	74	25	46	26	66	36	107	60	35	32	17	18	2	4
	Biogas Production	mL	3967	452	3736	179	4383	364	4933	276	4110	397	3901	277	3810	298	3913	257	3910	274
	Methane Content	%	51	1	51	0.5	51	0	51	0	50.5	0.5	50	0	50	0.5	49.5	0.5	48	0
	Hydrolysis Efficiency	%	50.0	0.6	46.8	3.9	54.2	3.7	62.9	1.7	50.3	0.2	54.0	0.0	48.7	0.0	50.6	0.0	48.9	0.0
	Acidogenesis Efficiency	%	52.3	6.9	46.9	2.3	57.8	4.3	63.0	3.6	53.3	5.4	50.6	3.2	49.4	3.9	48.9	2.2	47.5	3.0
	Methanogenesis Efficiency	%	51.0	5.8	46.5	2.2	55.5	4.6	62.5	3.5	52.1	5.0	48.5	3.4	49.9	4.1	48.2	2.9	47.1	3.5
R5	Acetate Concentration	mg /L COD	95	33	64	22	87	27	69	37	38	22	35	22	31	18	16	17	5	11
	Propionate Concentration	mg /L COD	510	345	61	40	80	20	63	40	52	46	61	33	17	21	2	6	0	0
	Biogas Production	mL	3805	392	3762	345	4161	189	4351	196	4095	204	4035	623	4140	370	3919	141	3971	319
	Methane Content	%	52.5	0.5	52	0.5	51	0	51	0	50.5	0.5	50	0	50	0.8	51.5	0.5	49	0
	Hydrolysis Efficiency	%	50.9	0.5	48.7	3.9	52.8	3.7	54.2	1.7	51.1	0.2	60	0	53.6	0	52.4	0	49.9	0
	Acidogenesis Efficiency	%	54.9	8.4	49.3	4.5	57.8	4.3	56.4	3.0	52.5	2.7	50.9	8.3	52.2	3.6	50.9	1.5	49.0	2.9
	Methanogenesis Efficiency	%	50.0	5.2	48.5	4.4	52.7	2.4	55.1	2.5	51.9	2.6	50.1	7.7	54.7	4.8	49.8	1.7	48.3	3.8

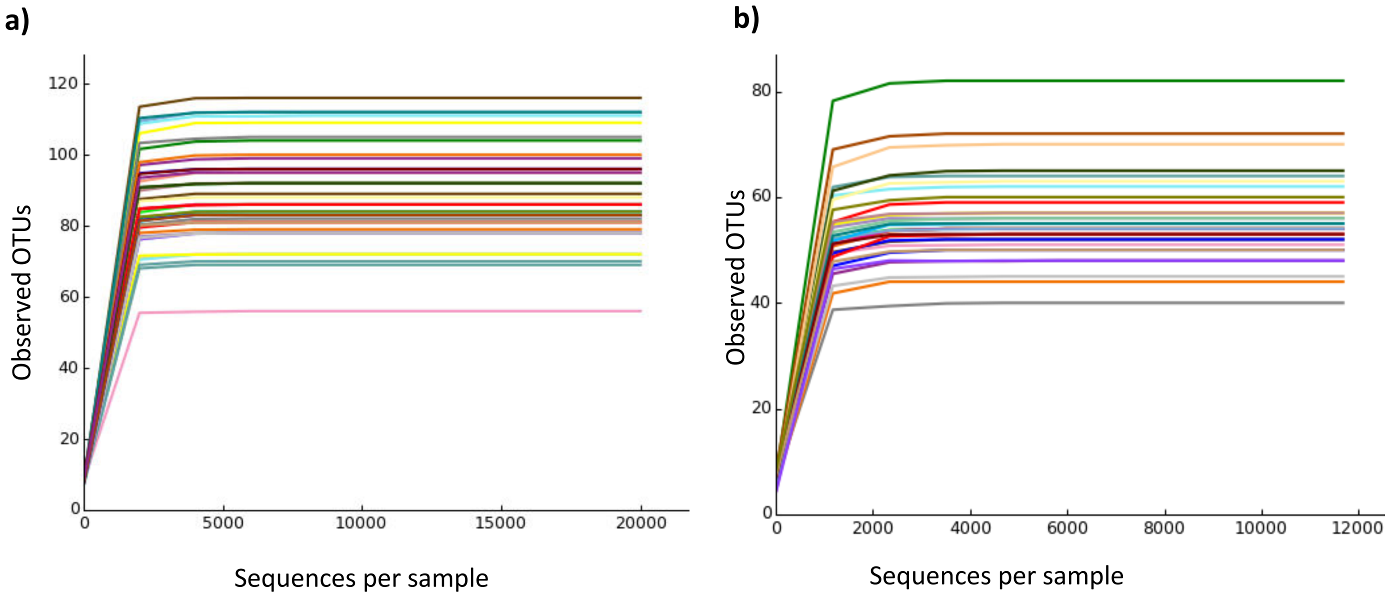

Fig. S1 Rarefaction curves showing the number of observed OTUs against sampling depth of each of the a)bacterial and b)archaeal samples

Supplementary Table S2 The number of reads per sample that was obtained by next generation sequencing.

Sampling points	Reactor	Bacterial reads	Archaeal reads
	R1	206686	51312
	R2	183824	71481
	R3	190662	38170
	R4	73253	32974
P0	R5	67189	12665
	R1	141518	21686
	R2	241952	30432
	R3	122191	17946
	R4	46220	8974
P1	R5	161299	13941
	R1	4748	2569
	R2	30187	17569
	R3	71988	30052
	R4	228725	47558
P2	R5	106020	27049
	R1	87630	7281
	R2	133360	22089
	R3	75941	16452
	R4	98322	31967
P3	R5	1015	1000<
	R1	4672	1029
	R2	168718	23809
	R3	138606	23555
	R4	122813	14585
P4	R5	74325	3739
	R1	191092	71665
	R2	220401	40089
	R3	62691	6807
	R4	65146	3279
P5	R5	40541	67287
	R1	418163	1000<
	R2	413256	170459
	R3	322267	57700
	R4	165088	17538
P6	R5	77314	1000<
	R1	21270	3459
	R2	50424	11688
	R3	185568	29807
	R4	270967	31139
P7	R5	229511	25456
	R1	220733	17580
	R2	159008	25149
	R3	376954	29153
	R4	121302	8229
P8	R5	133203	1000<

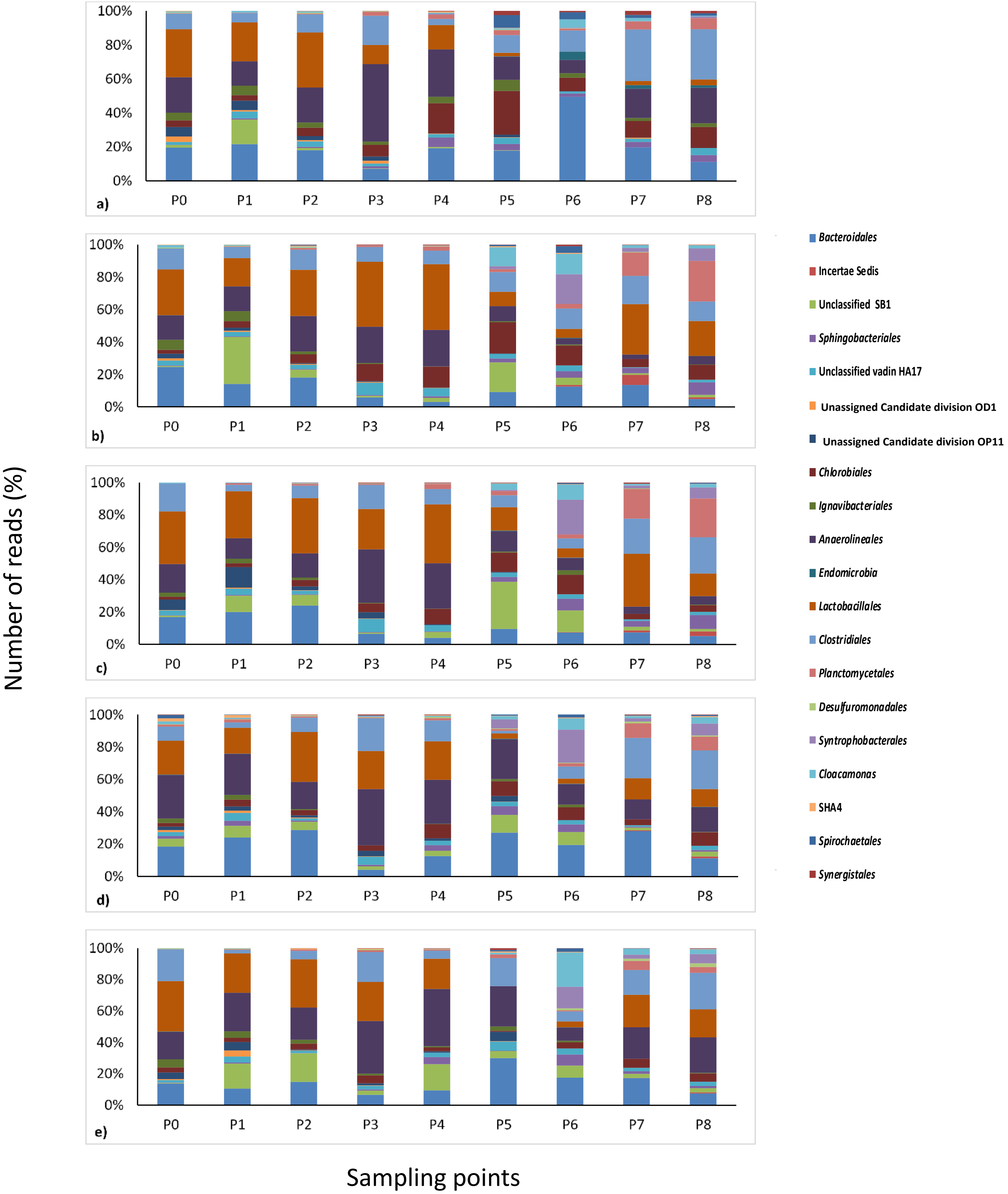

Fig. S2 Relative abundance of the bacterial community in the reactors a) R1 (control), b) R2 (inhibition), c) R3 (Ca addition), d) R4 (enzyme addition) and e) R5 (Ca and enzyme addition). Only the orders were presented that were abundant at a relative abundance >1 % in at least one sample. The term unassigned was used to indicate the bacterial groups that were not classified at order level.

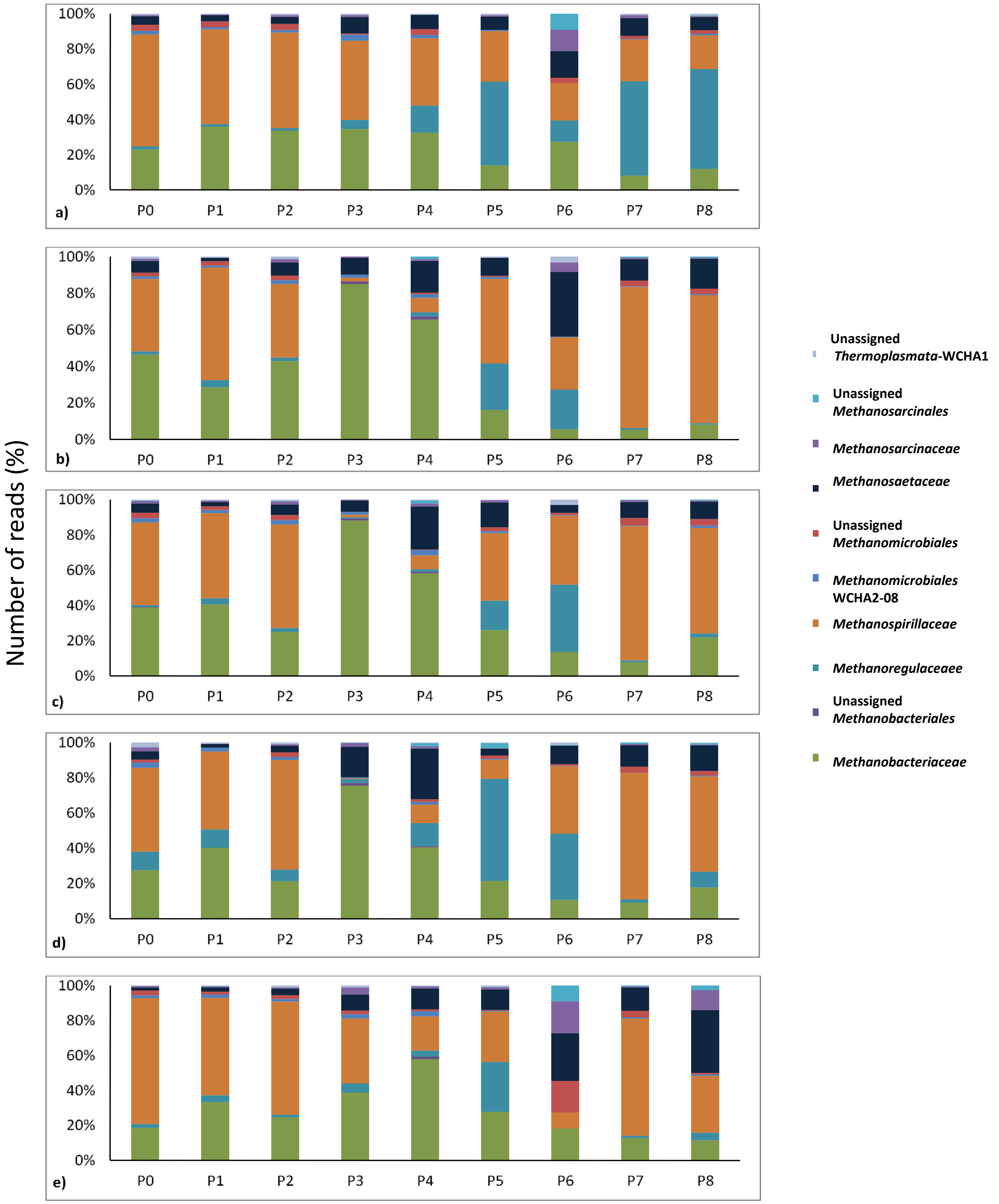

Fig. S3 Relative abundance of the archaeal community in the reactors a) R1 (control), b) R2 (inhibition), c) R3 (Ca addition), d) R4 (enzyme addition) and e) R5 (Ca and enzyme addition). Only the orders were presented that were abundant at an the relative abundance>1 % in at least one sample. The term unassigned was used to indicate the bacterial groups that were not classified at order level.

Supplementary Table S3 Correlation matrix between operational data and the bacterial orders. Negative correlations (red) and positive correlations (green) are shown. Correlations were determined by the two- tailed Spearman's Rank Order Correlations statistics.

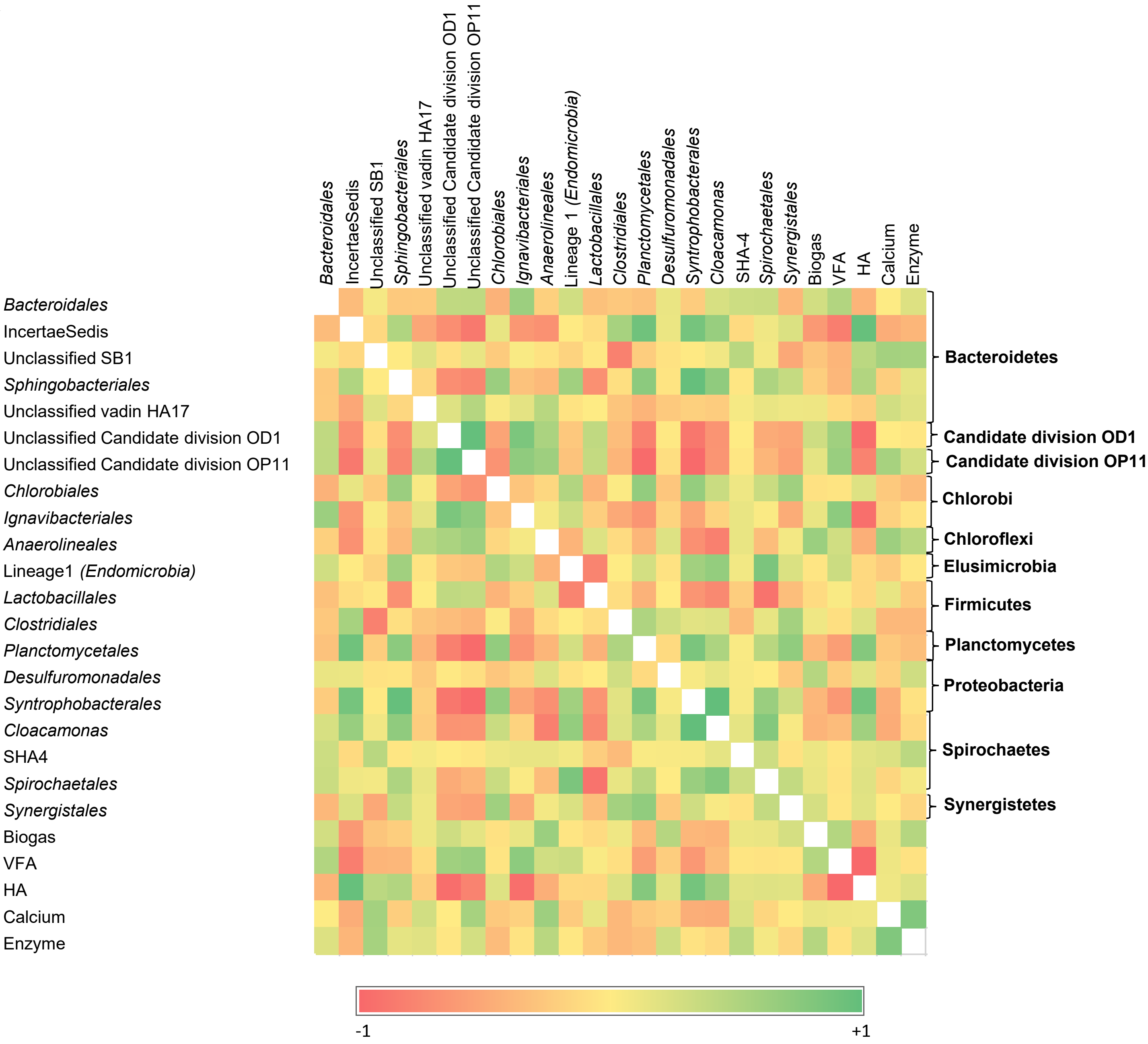

Supplementary Table S4 Correlation matrix between operational data and the archaeal families. Negative correlations (red) and positive correlations (green) are shown. Correlations were determined by the two- tailed Spearman's Rank Order Correlations statistics.

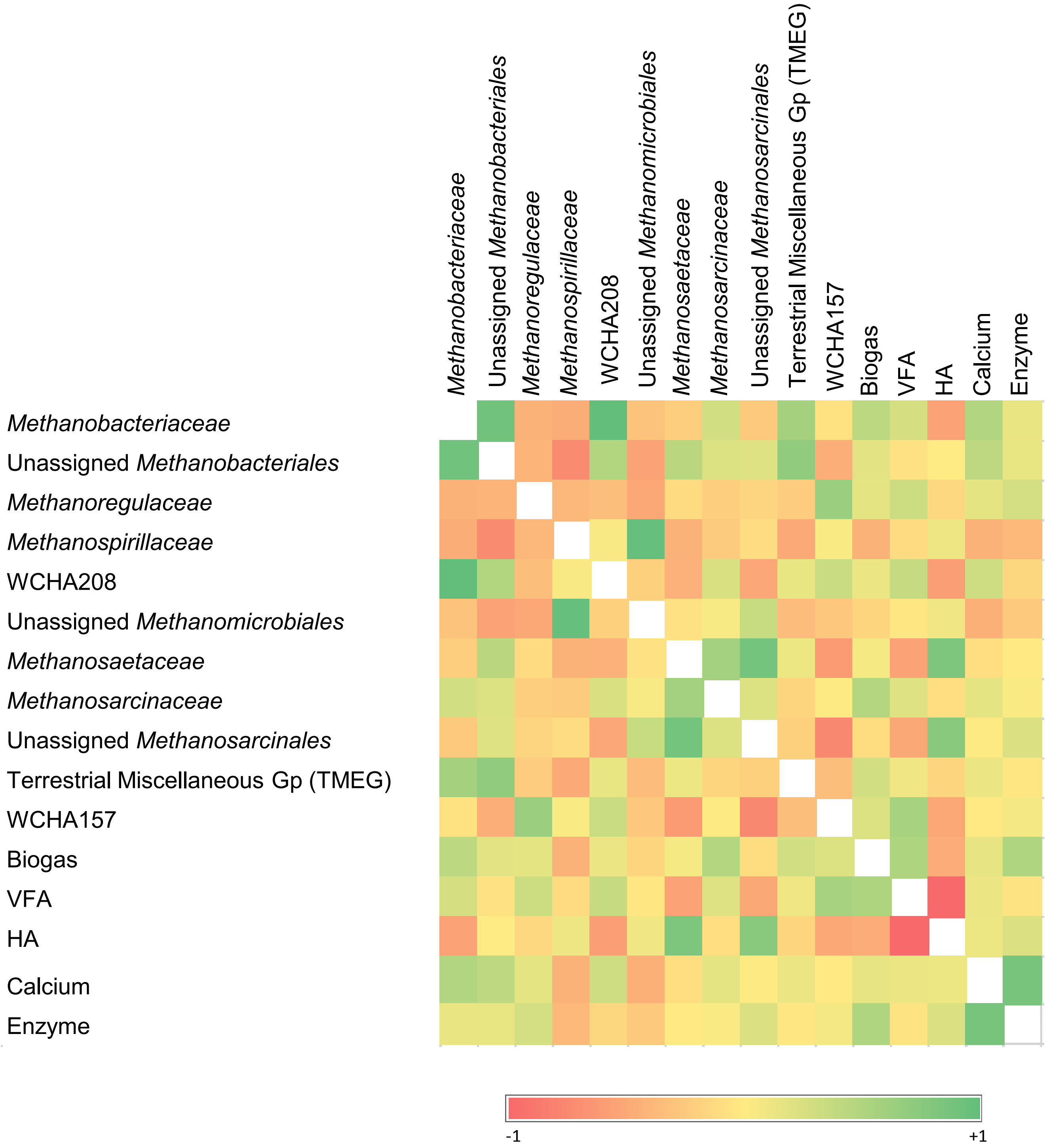

$$H(\%) = \frac{CODm,\, t{=}x + CODs,t{=}x - CODs,t{=}0}{COD\ total\ ,t{=}0} * 100 \qquad (1)$$

$$A\ (\%)\ = \frac{CODm\ ,\text{t=x} + CODv,t{=}x}{COD\ total\ ,t{=}0} * 100 \qquad (2)$$

$$M\ (\%)\ = \frac{CODm\ ,t{=}x}{COD\ total\ ,t{=}0} * 100 \qquad (3)$$

Formula S1: Formulas to calculate Hydrolysis (H), Acidogenesis (A) and Methanogenesis (M) efficiencies. CODm, t=x is the concentration of methane represented in COD (t=time; x=sampling time). CODs, t=x is the concentration of soluble COD at t=x, COD_V, t=x is the concentration of VFAs at t=x and CODtotal, t=0 is the total COD added at the beginning of each experiment.
